# The expression profile of type 1 equilibrative nucleoside transporter in aged C57BL/6J mouse brain: ENT1 expression in aged mouse brain

**DOI:** 10.3724/abbs.2025127

**Published:** 2025-07-28

**Authors:** Xiao-Yuan Zhang, Ziteng Ma, Ya-Han Jin, Zhimin Long, Qing Tang, Guiqiong He, Yun-Fang Jia

**Affiliations:** 1 Center for Neuroscience Research School of Basic Medical Sciences Chongqing Medical University Chongqing 400016 China; 2 Department of Anatomy School of Basic Medical Sciences Chongqing Medical University Chongqing 400016 China; 3 Department of Physiology School of Basic Medical Sciences Chongqing Medical University Chongqing 400016 China; 4 Key Laboratory of Major Brain Disease and Aging Research (Ministry of Education) Chongqing Medical University Chongqing 400016 China

**Keywords:** equilibrative nucleoside transporter 1, adenosine, brain, aging, cerebral cortex

## Abstract

The type 1 equilibrative nucleoside transporter (ENT1) is essential for regulating extracellular adenosine levels and has been implicated in various psychiatric disorders. While previous studies have investigated the expression of ENT1 in fetal and neonatal brains, its neuroanatomical distribution in adult and aged mouse brains has not been fully elucidated. Therefore, in this study, we utilize immunohistochemistry and double-immunofluorescence techniques to map the expression of ENT1 across various brain regions. Our findings demonstrate that ENT1-positive cells are widely distributed throughout the brain, with particularly high expression observed in the cerebral cortex and hippocampus. ENT1 is expressed predominantly in neurons, particularly in cholinergic, dopaminergic and glutamatergic neurons. Furthermore, ENT1 is localized primarily to the mitochondria and lysosomes and is expressed to a lesser extent in the Golgi apparatus and endoplasmic reticulum. Notably, we find an age-dependent increase in ENT1 expression in the cerebral cortex, suggesting a potential role in age-related cognitive functions. This study highlights the regionally specific expression of ENT1 in the brain, providing a new morphological basis for understanding its potential roles in brain physiology. Additionally, given its involvement in neurotransmitter regulation, ENT1 may have important implications for neurological and psychiatric disorders. This work lays the groundwork for future studies exploring the implications of ENT1 in neurodegenerative diseases and other aging-related brain disorders.

## Introduction

Adenosine is a purine nucleoside that can be produced both intracellularly and extracellularly through adenosine triphosphate (ATP) metabolism, and it plays a role as a neuromodulator, influencing various processes in the brain, such as sleep-wake cycles, neuroprotection, and neurotransmitter release regulation [ 1, 2]. Dysregulated adenosine signaling has been linked to multiple neurological conditions, including disturbances in sleep, pain, stroke, epilepsy, neurodegenerative diseases, and psychiatric disorders [ 3– 6]. Despite extensive studies on adenosine’s function, the origins of extracellular adenosine in the brain remain unclear.

Adenosine levels in the central nervous system (CNS) are regulated not only by the breakdown of ATP from cells but also by two equilibrative nucleoside transporters (ENTs), ENT1 and ENT2, with ENT1 having the highest affinity for adenosine [7]. ENT1 controls adenosine transport across cell membranes, balancing intracellular and extracellular levels [8]. A study using a genetically encoded adenosine sensor (GRABAdo) revealed that adenosine is gradually released from the postsynaptic membrane through an ENT1-dependent process, which suggests that adenosine may act as a retrograde signal to regulate presynaptic activity [9]. This retrograde signaling highlights ENT1’s role in modulating adenosine availability to its receptors and its impact on downstream pathways crucial for CNS function [10]. ENT1 has been implicated in a range of nervous system conditions, including anxiety, circadian rhythm regulation, alcohol addiction, Huntington’s disease, and Alzheimer’s disease (AD) [ 11– 16]. Although the precise mechanisms by which ENT1 influences these disorders have not been fully elucidated, its regulatory effect on adenosine signaling suggests significant therapeutic potential.

The distribution pattern of ENT1 in the mammalian brain is particularly intriguing, given its potential to reveal regional variations in adenosine signaling and associated therapeutic implications. Mapping ENT1 across various brain regions is a critical step toward understanding its physiological roles and the neuropathology of various disorders. The distribution of ENT1 in the brain has been shown using *in situ* hybridization, quantitative real-time PCR (qRT-PCR), northern blot analysis, western blot analysis, autoradiographic and membrane binding techniques [ 17– 20]. However, all of these techniques are limited in terms of the localization of transporters at the cellular and subcellular levels. To obtain more precise information on the distribution of ENT1, we utilized immunohistochemistry (IHC) and immunofluorescence (IF) techniques to map ENT1 spatial expression in the brain. By combining western blot analysis and qRT-PCR, we further explored the temporal expression of ENT1 by analyzing the difference between young and aged patients to better understand its functional significance in CNS homeostasis and pathology.

## Materials and Methods

### Mice

Male C57BL/6J mice aged 1, 3, 6, 9, or 12 months were purchased from the Animal Center of Chongqing Medical University and used in this study. For the analysis of ENT1 expression via IHC and IF staining, we used 9-month-old mice, with 3 mice in each group. To examine ENT1 expression across different ages, we used young adult (approximately 3 months old) and middle-aged (approximately 9 months old) mice, with three mice in each group. At least 3–5 slices per mouse were analyzed for each index. The mice were group-housed (4–5 mice per cage) under standard specific pathogenfree (SPF) conditions with freely accessible water and a standard chow diet and kept under controlled lighting with a 12-h/12-h light (500 lux)/dark (< 0.5 lux) cycle in a temperature (22–24°C)- and humidity (50%)-controlled environment. All experiments were conducted following protocols approved by the Chongqing Medical University Animal Ethics Committee (or Institutional Animal Care and Use Committee, IACUC-CQMU-2024-0500).

### Brain tissue preparation

The mice were anaesthetized with 2% isoflurane (R510-22; RWD, Shenzhen, China), and the cardiac apex was carefully exposed. After the syringe needle was inserted into the cardiac apex, the right atrial appendage was cut open. At this point, the heart should be pulsating. Saline was slowly and uniformly injected. The injection was stopped when the entire blood in the body was completely flushed out, indicating that the mice were fully deceased and that the head was disconnected with scissors. The brains were rapidly removed, fixed in 4% paraformaldehyde (BL539A; Biosharp, Hefei, China) for 24 h at 4°C, and then dehydrated with fractionated ethanol. After being embedded in paraffin wax, coronal sections (5 μm) were cut with a paraffin microtome (RM2016; Leica, Wetzlar, Germany) and then mounted on glass slides for subsequent IHC and IF staining experiments.

### IHC staining

The sections were deparaffinized in xylene 3 times (15 min each time) at room temperature and then rehydrated by passing through ethanol absolute, 85%, 75% ethanol, and distilled water for 5 min each in sequence. After being washed with PBS (AFIHC018; AiFang, Changsha, China), the antigens were retrieved from the sections with sodium citrate (pH 6.0) (AFIHC009; AiFang) for 20 min at 95°C, after which they were cooled to room temperature. After being washed with PBS, the sections were blocked with 3% H _2_O _2_ for 15 min without light for endogenous peroxidase activity. The sections were blocked with 5% bovine serum albumin (BSA) (G5001; Servicebio, Wuhan, China) for 30 min at room temperature and then incubated with the primary rabbit anti-ENT1 antibody (YT5031, 1:200; Immunoway, San Jose, USA) overnight at 4°C. After being washed with phosphate-buffered saline (PBS), the sections were incubated with an HRP-conjugated anti-rabbit secondary antibody (AFIHC003; AiFang) for 50 min at room temperature. After being washed with PBS, the sections were incubated with DAB (AFIHC004; AiFang), and when the positive cells were stained brownish yellow, this step was finished. Then, the sections were incubated with hematoxylin for approximately 1‒2 min and dehydrated by passing through 75%, 85% ethanol, ethanol absolute, butyl alcohol and xylene for 5 min each in sequence. When the sections were dry, they were covered with a coverslip with neutral gum. Finally, the sections were photographed with a Leica microscope (Leica). The statistical method we used was to count the number of ENT1-positive cells per slide observed under a 40× microscope.

### IF staining

A TSA (tyramide signal amplification) kit (AFIHC023; AiFang) was used in this method. The sections were deparaffinized, rehydrated, subjected to antigen repair, blocked and washed, and then incubated with the first primary antibodies including rabbit anti-Iba1 (10904-1-AP, 1:500; Proteintech, Wuhan, China), mouse anti-GFAP (38014, 1:100; SAB, Greenbelt, USA), rabbit anti-MAP2 (17490-1-AP, 1:500; Proteintech), rabbit anti-PSD95 (20665-1-AP, 1:200; Proteintech), mouse anti-MBP (83683S, 1:500; CST, Danvers, USA), rabbit anti-AChE (AF5274, 1:100; Affinity Biosciences, Cincinnati, USA), rabbit anti-DAT (DF4529, 1:100; Affinity Biosciences), rabbit anti-VGluT1 (DF13657, 1:100; Affinity Biosciences), rabbit anti-GAD65 (21760-1-AP, 1:500; Proteintech), rabbit anti-GM130 (A11408, 1:500; ABclonal, Wuhan, China), rabbit anti-Calnexin (A15631, 1:500; ABclonal), rabbit anti-LAMP1 (ab278043, 1:100; Abcam, Cambridge, UK), rabbit anti-TOMM20 (ET1609-25, 1:100; HUABIO, Hangzhou, China), and rabbit anti-COX IV (AF5468, 1:100; Affinity Biosciences) overnight at 4°C, After washing with PBST, the sections were incubated with the first HRP-conjugated secondary antibody for 30 min at room temperature, followed by TYR-520 (excitation light 490 nm) incubation for approximately 3–10 min. The sections were then subjected to repeated antigen repair, blocking and washing, and incubated with the second primary antibody rabbit anti-ENT1 (YT5031, 1:100; Immunoway) overnight at 4°C. After being washed with PBST, the sections were incubated with the second HRP-conjugated secondary antibodies for 30 min at room temperature, followed by incubation with TYR-570 (excitation light 550 nm) for approximately 3–10 min. After thorough rinsing with PBST, the slides were mounted using 4,6-diamidino-2-phenylindole (DAPI) for cell nuclear counterstaining. For isotype controls, we used species- and concentration-matched isotype controls for primary antibodies: rabbit IgG (98136-1-RR; Proteintech; 1:100 for AChE/DAT/VGluT1/LAMP1/TOMM20/COXIV/ENT1, 1:200 for PSD95, 1:500 for Iba-1/MAP2/GAD65/GM130/Calnexin) and mouse IgG2b (66360-3-Ig; Proteintech; 1:100 for GFAP, 1:500 for MBP). All other experimental conditions matched the primary antibody treatments. For negative controls, primary antibodies were omitted and replaced by antibody diluent (the buffer used to dilute the primary antibody), while all other experimental conditions remained unchanged. This included overnight incubation at 4°C with antibody diluent, washing steps, and the application of secondary antibodies (HRP-520, green and HRP-570, red) matched those used in the experimental groups.

Fluorescence images were captured using a Leica Thunder microscope system (Leica) with 40×/0.6 NA under identical exposure conditions across samples. Colocalization analysis was performed using the Coloc2 plugin in ImageJ 1.51a (NIH, Bethesda, USA) with the following specific parameters: Costes automatic thresholding was applied, and the analysis was restricted to region of interest (ROI) encompassing cellular compartments while excluding background areas. Pearson’s correlation coefficient (R) was calculated from these images using the following standardized interpretation: R > 0.5 indicates strong colocalization ( *P* < 0.01); 0.2 ≤ R ≤ 0.5 indicates moderate colocalization ( *P* < 0.05); and R < 0.2 indicates no significant correlation. For each experimental condition, we analyzed 3–5 randomly selected fields from 3 independent biological replicates, quantifying 3–5 cells per field. The final R values are presented as the mean ± SEM. All analyses were performed by a researcher who was blinded to the experimental conditions.

### Western blot analysis

The mice were anaesthetized with 2% isoflurane, and then the head was disconnected with scissors. The brains were rapidly removed without perfusion and then frozen at –80°C. The brain tissues were homogenized with RIPA lysis buffer (P0013B; Beyotime, Shanghai, China) containing 1% PMSF (P1045; Biosharp) and 1% phosphatase inhibitor (P1045; Beyotime). After centrifugation at 12,000 *g* for 30 min, the supernatant was collected, and the protein concentration was quantified using an enhanced BCA protein assay kit (P0010; Beyotime). Proteins (40 μg/lane) were separated by electrophoresis on 10% Tris-glycine polyacrylamide gels (Epizyme, Shanghai, China) and then transferred to polyvinylidene fluoride membranes. After being blocked with 5% BSA for 1 h at room temperature, the membrane was incubated with primary antibodies, including rabbit anti-ENT1 (DF8542, 1:1000, 50 kDa; Affinity Biosciences) or rabbit anti-GAPDH (AF7021, 1:5000, 37 kDa; Affinity Biosciences), overnight at 4°C. After being washed with TBST, the membranes were incubated with a fluorescence-conjugated anti-rabbit IgG secondary antibody (D30221-01, 1:10,000; LI-COR, Lincoln, USA). The immunoreactive bands were visualized with an Odyssey LI-COR infrared fluorescence imaging system. The relative protein levels were quantified via LI-COR image studio software and normalized to the level of GAPDH.

### Quantitative real-time PCR (qRT-PCR)

The mice were anaesthetized with 2% isoflurane, and then the head was disconnected with scissors. The brains were rapidly removed and then frozen at -80°C. Total RNA from the brain tissue was isolated using TRNzol Universal RNA Reagent (Y2206; TIANGEN, Beijing, China), and the concentration was determined with a Nano spectrophotometer (7541; Biodrop, Hangzhou, China). cDNA was obtained using a transcription system (RM21479; ABclonal), and the thermal cycling protocol for reverse transcription was as follows: 2 min at 37°C, 15 min at 55°C, and then 5 min at 85°C. qPT-PCR was conducted on a CFX96 Touch Real-Time system, C1000 Touch Thermal Cycler (Bio-Rad, Hercules, USA) using Universal SYBR Green Fast Mix (RM21203; ABclonal) and gene-specific primers (for the targeted *ENT1* gene: forward primer: 5′-AGAGTATCTGTGTCCCGGCT-3′, reverse primer: 5′-AAGAAACAGGCCACGGGAAT-3′; for the endogenous reference gene *GAPDH*: forward primer: 5′-TGCACCACCAACTGCTTAGC-3′, reverse primer: 5′-GGCATGGACTGTGGTCATGAG-3′). The thermal cycling protocol for qRT-PCR was 45 amplification cycles of 15 s at 94°C, followed by 10 s at 55°C, and then 30 s at 72°C. The relative mRNA expression levels were normalized to those of *GAPDH*. The percentage changes were quantified by subtracting the *GAPDH* Ct values from the Ct values for the gene of interest using the 2 ^–ΔΔCt^ method.

### Statistical analysis

We used the minimum number of mice needed to obtain statistically significant results. All the data are expressed as the mean ± SEM. Two-tailed unpaired Student’s *t* tests were used to compare the differences between two groups. Differences were considered statistically significant when *P* < 0.05. There was no sample size calculation performed, but all the data in this study conformed to normality (Shapiro-Wilk test). All the statistical analyses were performed via GraphPad Prism version 10.4.1 (GraphPad Software, La Jolla, USA).

## Results

### Distribution pattern of ENT1 in the mouse brain

**Figure 1:**
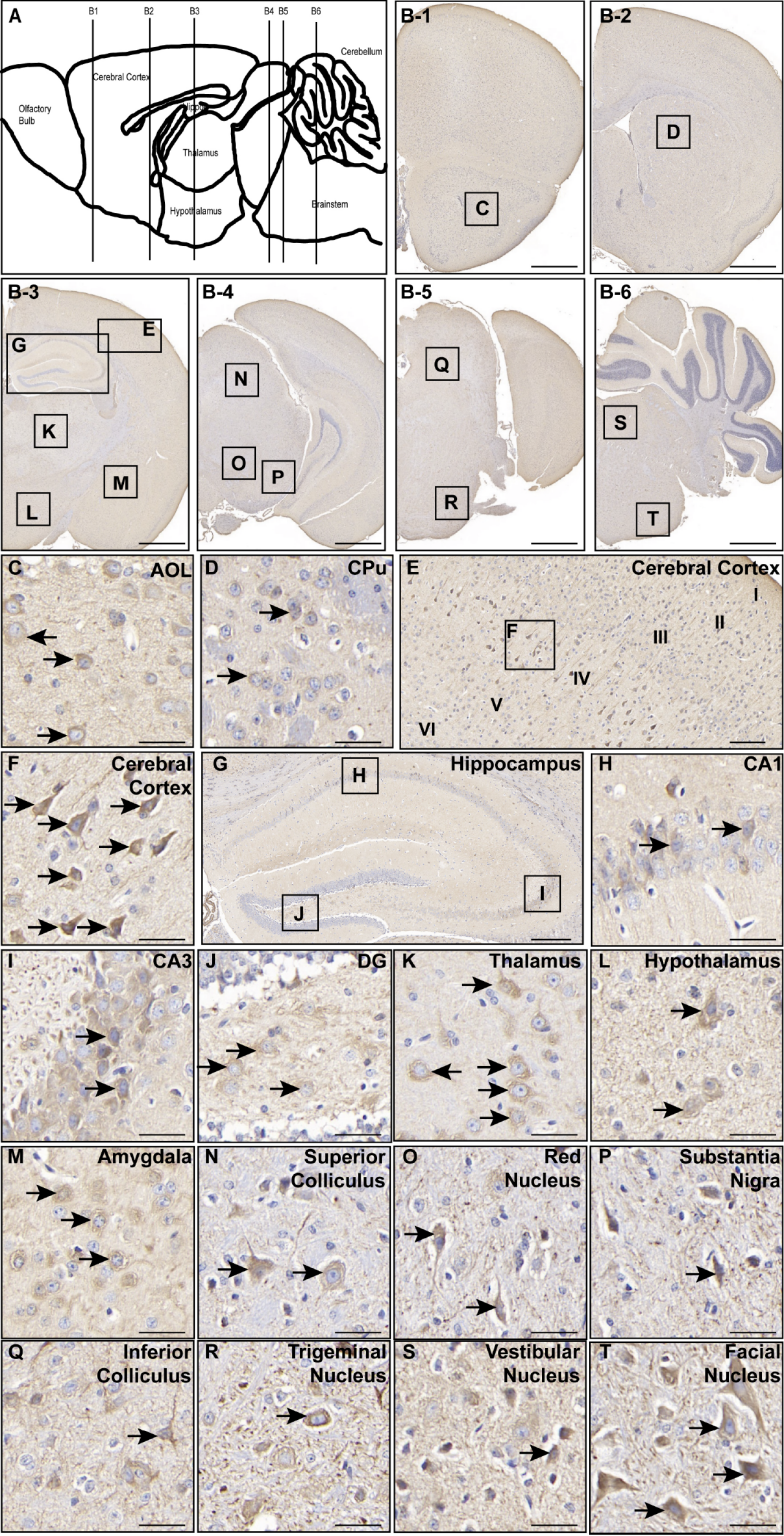
Figure 1 Distribution of ENT1 expression in the mouse central nervous system (A) A hand-drawn median sagittal section of the mouse brain indicating ENT1 distribution. (B1–B6) Representative coronal sections corresponding to sites marked by vertical lines in the sagittal section. (C–T) Magnified images of ENT1-positive regions from the coronal sections reveal areas with relatively high ENT1 expression, including the anterior olfactory nucleus, lateral part (C); striatum, CPu (D); cerebral cortex (E,F); hippocampus (G): CA1 (H), CA3 (I), DG (J); thalamus (K); hypothalamus (L); amygdala (M); superior colliculus (N); red nucleus (O); substantia nigra (P); inferior colliculus (Q); trigeminal nucleus (R); vestibular nucleus (S); and facial nucleus (T). Scale bars = 1 mm (B1–B6), 100 μm (E), 200 μm (G), and 40 μm (C,D,F,H–T). Blue indicates the nuclei, and brown indicates ENT1 immunopositive staining. We conducted experiments on 3 mice in this study, and representative images are shown.

**Figure 2:**
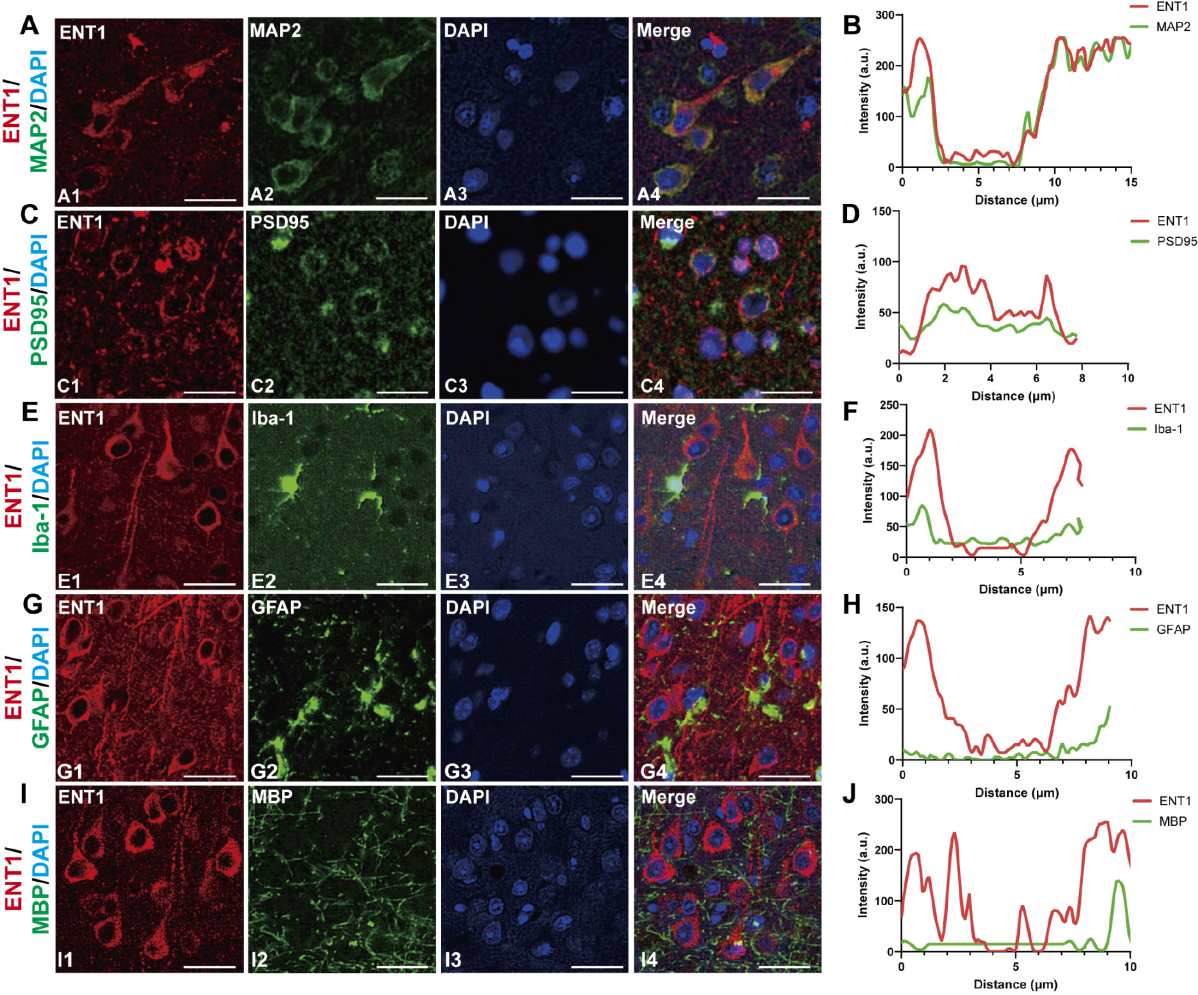
Figure 2 Cellular localization of ENT1 in the mouse brain (A) Representative images showing ENT1 staining with a neuronal marker (MAP2). (B) Statistical analysis of the co-localization of ENT1 (red) and MAP2 (green). (C) Representative images showing ENT1 staining of a neuronal marker (PSD95). (D) Statistical analysis of the co-localization of ENT1 (red) and PSD95 (green). (E) Representative images showing ENT1 staining of a microglial marker (Iba-1). (F) Statistical analysis of the co-localization of ENT1 (red) and Iba-1 (green). (G) Representative images showing ENT1 staining with an astrocytic marker (GFAP). (H) Statistical analysis of the co-localization of ENT1 (red) and GFAP (green). (I) Representative images showing ENT1 staining with an oligodendrocytic marker (MBP). (J) Statistical analysis of the co-localization of ENT1 (red) and MBP (green). Red fluorescence indicates ENT1 immunoreactivity, green fluorescence represents the respective cell markers, blue fluorescence labels DAPI (nuclei), and merged images highlight co-localization. Scale bar = 20 μm.

**Figure 3:**
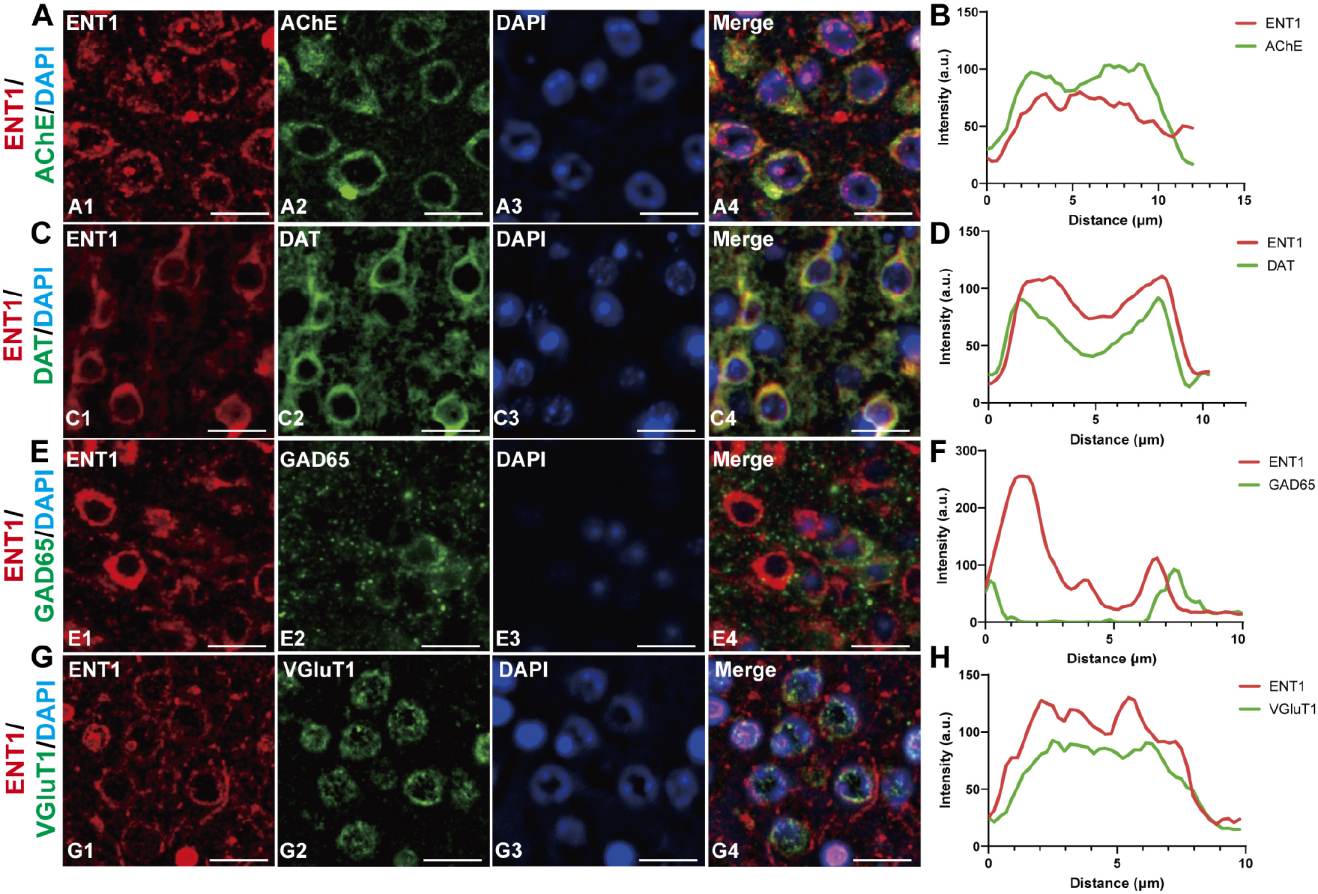
Figure 3 Neuronal type localization of ENT1 in the mouse brain (A) Representative images showing ENT1 staining of cholinergic neurons (AChE). (B) Statistical analysis of the co-localization of ENT1 (red) and AChE (green). (C) Representative images showing ENT1 staining of dopaminergic neurons (DAT). (D) Statistical analysis of the co-localization of ENT1 (red) and DAT (green). (E) Representative images showing ENT1 staining of GABAergic neurons (GAD65). (F) Statistical analysis of the co-localization of ENT1 (red) and GAD65 (green). (G) Representative images showing ENT1 staining of glutamatergic neurons (VGluT1). (H) Statistical analysis of the co-localization of ENT1 (red) and VGluT1 (green). Red fluorescence represents ENT1 immunoreactivity, green fluorescence indicates specific neuronal subtype markers, blue fluorescence labels DAPI (nuclei), and merged images display co-localization. Scale bar = 20 μm.

**Figure 4:**
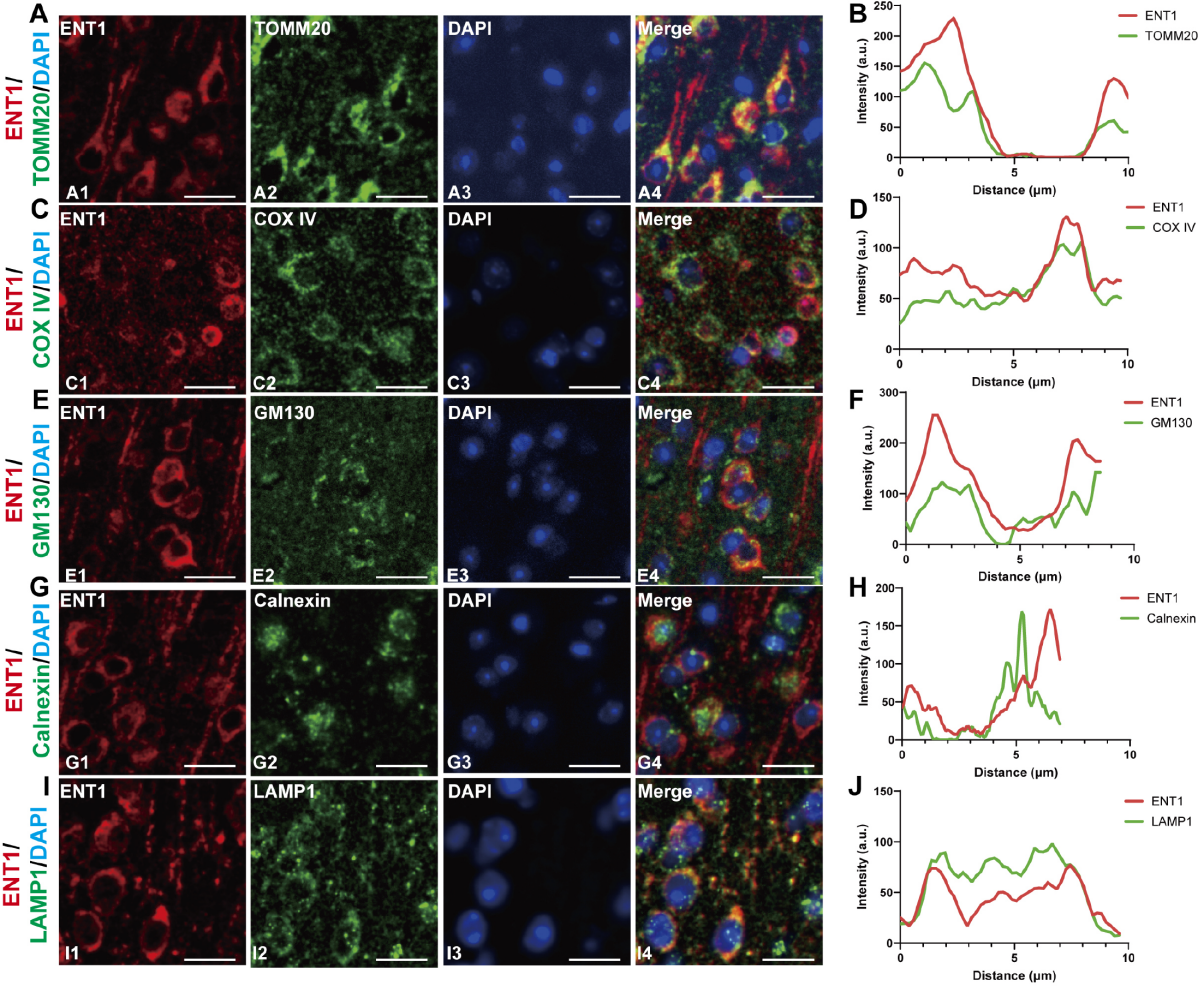
Figure 4 Subcellular localization of ENT1 in the mouse brain (A) Representative images showing ENT1 staining of subcellular organelle mitochondria (TOMM20). (B) Statistical analysis of the co-localization of ENT1 (red) and TOMM20 (green). (C) Representative images showing ENT1 staining of subcellular organelle mitochondria (COX IV). (D) Statistical analysis of the co-localization of ENT1 (red) and COX IV (green). (E) Representative images showing ENT1 staining of the Golgi complex (GM130). (F) Statistical analysis of the co-localization of ENT1 (red) and GM130 (green). (G) Representative images showing ENT1 staining in the endoplasmic reticulum (Calnexin). (H) Statistical analysis of the co-localization of ENT1 (red) and calnexin (green). (I) Representative images showing ENT1 staining with lysosomes (LAMP1). (J) Statistical analysis of the co-localization of ENT1 (red) and LAMP1 (green). Red fluorescence represents ENT1 immunoreactivity, green fluorescence indicates specific organelle markers, blue fluorescence labels DAPI (nuclei), and merged images display co-localization. Scale bar = 20 μm.

**Figure 5:**
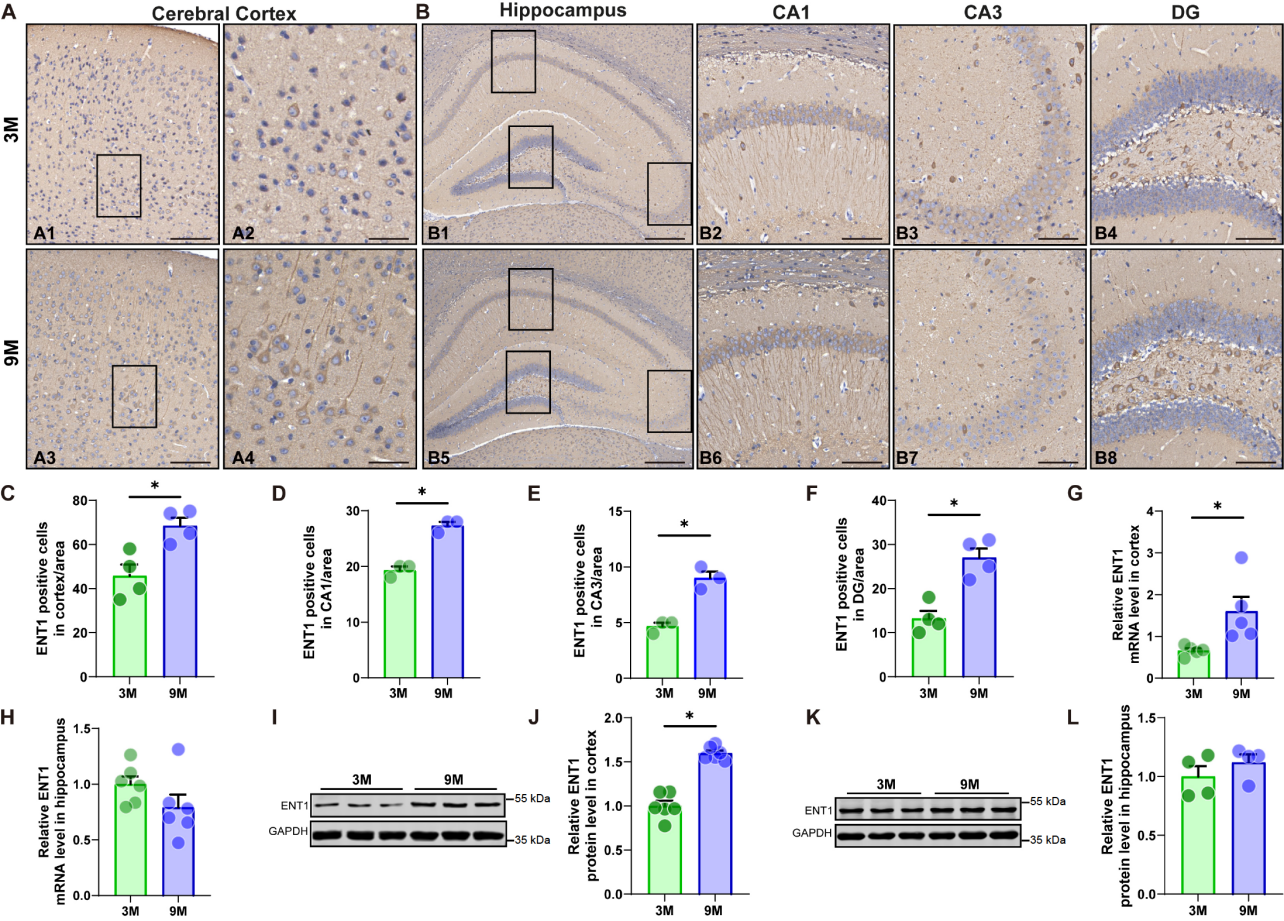
Figure 5 Age-dependent increase in ENT1 expression in the cortex and hippocampus (A) Representative images showing ENT1-positive cells in the cerebral cortex of 3-month-old (3M) (A1,A2) and 9M (A3,A4) mice using IHC. (B) Representative images showing ENT1-positive cells in the CA1, CA3, and DG of the hippocampus of 3M (B1–B4) and 9M (B5–B8) mice using IHC. (C) Quantification of ENT1-positive cells per unit area in the cortex of 3M and 9M mice via IHC. (D) Quantification of ENT1-positive cells per unit area in the CA1 region of the hippocampus of 3M and 9M mice via IHC. (E) Quantification of ENT1-positive cells per unit area in the CA3 region of the hippocampus of 3M and 9M mice via IHC. (F) Quantification of ENT1-positive cells per unit area in the DG of the hippocampus of 3M and 9M mice via IHC. The statistical method we used was to count the number of ENT1-positive cells observed under a 40× microscope. (G) ENT1 mRNA levels in the cortices of 3M and 9M mice were evaluated using real-time qPCR. (H) ENT1 mRNA levels in the hippocampi of 3M and 9M mice were evaluated using real-time qPCR. (I) ENT1 protein bands in the cerebral cortex of 3M and 9M mice were evaluated using western blot analysis. (J) Quantification of ENT1 protein levels relative to those of GAPDH in the cerebral cortex of 3M and 9M mice via western blot analysis. (K) ENT1 protein bands in the hippocampi of 3M and 9M mice were evaluated using western blot analysis. (L) Quantification of ENT1 protein levels relative to those of GAPDH in the hippocampi of 3M and 9M mice via western blot analysis. Scale bar = 150 μm (A1,A3), 300 μm (B1,B5), and 60 μm (A2,A4,B2–B8). n = 3–6 mice per group; *P < 0.05 indicates significant differences. Data are presented as the mean ± SEM.

We first employed IHC analysis to assess the expression levels of ENT1 in adult C57BL/6J mice at five different ages: 1, 3, 6, 9, and 12 months. The IHC results revealed that ENT1 expression was increased with age, reaching its highest level in 9-month-old mice in the cerebral cortex and hippocampus ( Supplementary Figure S1), regions prominent in ENT1-positive staining. On the basis of these findings, we focused on 9-month-old mice for subsequent detailed investigations into the distribution of ENT1 expression. Next, to investigate the regional distribution pattern of ENT1, we performed IHC staining on the brains of adult C57BL/6J mice using an ENT1 antibody. Six coronal sections of the mouse brain from anterior to posterior were selected to stain ENT1-positive cells ( Figure 1A), and each section was made of 5 consecutive slices. The sections included the olfactory bulb layer ( Figure 1B-1, Bregma: 2.34 mm; Interaural: 6.14 mm); the striatum layer ( Figure 1B-2, Bregma: 0.02 mm; Interaural: 3.82 mm); the layer encompassing the hippocampus, thalamus, hypothalamus, and amygdala ( Figure 1B-3, Bregma: –1.70 mm; Interaural: 2.10 mm); the layer encompassing the red nucleus and substantia nigra ( Figure 1B-4, Bregma: –4.04 mm; Interaural: –0.24 mm); and two layers of the brainstem ( Figure 1B-5, Bregma: –5.02 mm; Interaural: –1.22 mm; Figure 1B-6, Bregma: –6.36 mm; Interaural: –2.56 mm). High-resolution images revealed subregion-specific differences in ENT1-positive cell density throughout the brain ( Figure 1C–T). Specifically, in the anterior olfactory nucleus, ENT1-positive cells were especially concentrated in the lateral part ( Figure 1C); in the striatum, ENT1-positive cells were located primarily in the caudate shell nucleus ( Figure 1D). Within the cerebral cortex, ENT1-positive cells were mainly found in the outer granular layer (layer II) and inner granular layer (layer IV), with particularly strong expression in layer IV and weak or nearly no expression in other cortical layers ( Figure 1E). The staining revealed membrane localization around the nuclei of the cells, suggesting that ENT1 is expressed as a transmembrane protein ( Figure 1F). In the hippocampus ( Figure 1G), ENT1-positive cells were concentrated in pyramidal cells in the CA1 region ( Figure 1H), CA3 pyramidal cells ( Figure 1I), and mossy cells in the dentate gyrus (DG) area ( Figure 1J). Notably, ENT1 was more prominent in the thalamus ( Figure 1K), hypothalamus ( Figure 1L), and amygdala ( Figure 1M). Weaker ENT1 staining was detected in regions such as the erythrocytic nucleus, substantia nigra, trigeminal nucleus, and facial nucleus ( Figure 1N–T). Together, these results suggest that ENT1 is widely distributed throughout the mouse brain and is particularly concentrated in the cortex, hippocampus, thalamus, and hypothalamus, which implies that ENT1 may play important physiological roles in these brain regions.

### Cell type expression of ENT1

Given that ENT1 expression is particularly prominent in the cerebral cortex, we focused our subsequent experiments on this region. To determine the cell-type specificity of ENT1 expression in the mouse brain, we conducted double-IF staining using an ENT1 antibody alongside the following markers: neurons, MAP2 and PSD95; microglia, Iba-1; astrocytes, GFAP; and oligodendrocytes, MBP. We revealed that ENT1 expression was most prominent in MAP2-positive cells ( Figure 2A). The quantitative fluorescence analysis demonstrated co-localization between ENT1 and MAP2, as evidenced by a Pearson’s correlation coefficient (R = 0.818 ± 0.02764; Figure 2B). While MAP2 immunoreactivity was primarily observed in neuronal dendrites, detectable signals were also identified in somatic regions and minimally in axons [ 21– 24]. This spatial correlation indicated ENT1 localization in both neuronal dendrites and somas. Additionally, our analysis revealed that ENT1 colocalized with PSD95-positive staining (R = 0.52 ± 0.0204; Figure 2C,D), demonstrating its synaptic localization. However, ENT1 did not co-localize with Iba-1-positive microglia, as shown by the non-overlapping fluorescence intensity trends and a Pearson’s R value of 0.286 ± 0.02993, indicating a negligible association ( Figure 2E,F). Similarly, ENT1- and GFAP-positive astrocytes rarely overlapped, with Pearson’s coefficient analysis further confirming minimal associations (R = 0.098 ± 0.03555; Figure 2G,H). Finally, while MBP staining revealed broad oligodendrocyte distribution, there was no significant overlap with ENT1, as confirmed by fluorescence intensity analysis and a low Pearson’s coefficient (R = 0.106 ± 0.02522; Figure 2I,J). These findings suggest that ENT1 is predominantly expressed in the dendrites and soma of neurons, highlighting its potential role in regulating neuronal activity.

### Neurotransmitter-type neuron localization of ENT1

Given the role of ENT1 in regulating soma dendritic adenosine release [9], it is essential to determine the specific neuronal subtypes that express ENT1. To clarify ENT1 expression across various neurotransmitter systems, we performed double-IF staining using antibodies against ENT1 and neurotransmitter-specific markers for cholinergic, dopaminergic, GABAergic, and glutamatergic neurons. Using an antibody against acetylcholinesterase (AChE), a membrane-localized enzyme marker for cholinergic neurons, we observed clear co-expression of ENT1 and AChE ( Figure 3A). Further Pearson correlation analysis revealed significant co-localization (R = 0.604 ± 0.03473; Figure 3B). We next evaluated dopamine transport (DAT), a marker for dopaminergic neurons, and demonstrated ENT1 distribution in dopaminergic neurons ( Figure 3C), with a Pearson coefficient of R = 0.806 ± 0.01568 ( Figure 3D). However, ENT1 did not co-localize with glutamic acid decarboxylase 65 (GAD65), which is a GABAergic neuron marker ( Figure 3E), and exhibited a near-random correlation (R = 0.074 ± 0.01631; Figure 3F). Notably, ENT1 was co-expressed with vesicular glutamate transporter 1 (VGluT1) in glutamatergic neurons (R = 0.642 ± 0.03338; Figure 3G,H). These results indicate that ENT1 expression is highly specific to cholinergic, dopaminergic, and glutamatergic neurons where it likely modulates their physiological functions, but is nearly absent in GABAergic neurons.

### Subcellular localization of ENT1

To investigate the intracellular localization of ENT1, we performed double-IF staining with organelle-specific markers. We first assessed mitochondrial localization by co-staining with translocase of outer mitochondrial membrane 20 (TOMM20), a marker for the mitochondrial outer membrane. Strong co-localization with ENT1 was observed ( Figure 4A), which was supported by a high Pearson’s coefficient of R = 0.762 ± 0.02223 ( Figure 4B). To further validate these findings, we then employed cytochrome C oxidase subunit IV (COX IV), an inner mitochondrial membrane marker, which also significantly co-localized with ENT1 ( Figure 4C). Quantitative analysis confirmed this overlap (R = 0.58 ± 0.02121; Figure 4D), confirming the presence of ENT1 in the mitochondria. Next, we examined the presence of ENT1 in other organelles. Costaining with GM130 (Golgi matrix protein 130) revealed partial localization to the Golgi apparatus (R = 0.45 ± 0.01817; Figure 4E,F). Similarly, modest but consistent overlap was observed with the endoplasmic reticulum marker calnexin (R = 0.384 ± 0.02249; Figure 4G,H). Strikingly, ENT1 strongly colocalized with the lysosomal marker LAMP1 (lysosomal-associated membrane protein 1; R = 0.706 ± 0.01503; Figure 4I,J), suggesting significant lysosomal accumulation. Collectively, these results demonstrate that ENT1 is localized to multiple intracellular compartments, with a predominant distribution in mitochondria and lysosomes and a secondary presence in the Golgi apparatus and endoplasmic reticulum. These findings imply potential functional roles for ENT1 across distinct subcellular domains.

To account for potential nonspecific binding mediated by the antibody structure or isotype properties, we performed rigorous isotype controls using species-matched immunoglobulins at concentrations identical to those of primary antibodies. No positive staining was observed for any isotype controls: rabbit IgG at a 1:100 dilution (for primary antibodies against AChE, DAT, VGluT1, LAMP1, TOMM20, COX IV, and ENT1; Supplementary Figure S2A); rabbit IgG at a 1:200 dilution (for PSD95; Supplementary Figure S2B); rabbit IgG at a 1:500 dilution (for Iba-1, MAP2, GAD65, GM130, and calnexin; Supplementary Figure S2C); mouse IgG2b at a 1:100 dilution (for GFAP; Supplementary Figure S2D); or mouse IgG2b at a 1:500 dilution (for MBP; Supplementary Figure S2E). We further confirmed that the observed signals were specific to primary antibody binding rather than nonspecific secondary antibody interactions. We omitted primary antibodies while all other experimental conditions remained unchanged and showed that neither HRP-520 ( Supplementary Figure S2F) nor HRP-570 ( Supplementary Figure S2G) exhibited detectable fluorescence, confirming the specificity of our staining and the reliability of the antibodies. These results confirm the absence of nonspecific binding attributable to antibody properties, validating the specificity of our immunostaining.

### Age-dependent alterations in ENT1 expression

ENT1 plays a role in neurotransmitter modulation by regulating adenosine levels, which are crucial for neural signaling and protection against excitotoxicity. Changes in its expression with age could affect various cognitive and neuroprotective mechanisms in the brain. To investigate the temporal expression of ENT1 in the cerebral cortex and hippocampus, regions closely associated with cognitive function, we performed IHC staining, qRT-PCR, and western blot analysis to examine ENT1 expression in young adult (approximately 3 months old) and middle-aged (approximately 9 months old) mice. The IHC results revealed more pronounced ENT1-positive staining in the cerebral cortex ( Figure 5A) and hippocampus ( Figure 5B) of middle-aged mice than in those of young adult mice. The ratio of ENT1-positive cells to total cells in each region was significantly greater in the cerebral cortex of middle-aged mice than in that of young adult mice ( Figure 5C; two-tailed unpaired *t* test, *t*_6_ = 3.621, *P* = 0.0111). Similarly, ENT1 expression in CA1, CA3 and DG, which are brain regions related to cognitive function, was significantly higher in the hippocampi of middle-aged mice than in those of young adult mice ( Figure 5D–F; two-tailed unpaired *t* test, 5D: *t*_4_ = 8.485, *P* = 0.0011; 5E: *t*_4_ = 6.5, *P* = 0.0029; 5F: *t*_6_ = 5.056, *P* = 0.0023). Moreover, qRT-PCR analysis confirmed increased *ENT1* mRNA expression in the cortex of middle-aged mice compared with young adult mice ( Figure 5G; two-tailed unpaired *t* test, *t*_6_ = 3.579, *P* = 0.0117). However, the qRT-PCR results revealed no significant difference in *ENT1* mRNA levels in the hippocampus between middle-aged and young adult mice ( Figure 5H; two-tailed unpaired *t* test, *t*_8_ = 0.1113, *P* = 0.9141). The protein level of ENT1 was significantly higher in the cortex of middle-aged mice than in that of young adult mice ( Figure 5I,J; two-tailed unpaired *t* test, *t*_10_ = 9.004, *P* = 0.0001). In contrast, no significant difference in ENT1 protein levels was detected in the hippocampus between middle-aged and young adult mice ( Figure 5K,L; two-tailed unpaired *t* test, *t*_6_ = 1.082, *P* = 0.3207). While IHC analysis revealed region-specific increases in ENT1-positive staining within discrete hippocampal subfields, qRT-PCR and western blot analysis of whole hippocampal lysates failed to detect significant changes in overall ENT1 expression. Together, these results suggest that ENT1 expression is significantly higher in middle-aged mice than in young adult mice.

## Discussion

We revealed that ENT1 is predominantly expressed in the cerebral cortex and hippocampus, particularly in neurons, with a strong affinity for cholinergic, dopaminergic and glutamatergic neurons and is localized primarily in the mitochondria and lysosomes. Additionally, we observed an age-related increase in ENT1 expression in the cortex, suggesting its involvement in age-dependent cognitive functions. These findings underscore ENT1’s potential influence on physiological functions, supporting its relevance in brain homeostasis and age-related neural processes.

Previous studies have demonstrated that ENT1 is widely expressed across various brain regions in both humans and rats, including areas such as the hippocampus, cerebellum, cortex, striatum, and certain parts of the thalamus, hypothalamus, and brainstem [ 17 – 20, 25]. Our findings in the mouse brain are consistent with this distribution, highlighting ENT1’s regionally variable expression and suggesting specialized roles within distinct neurochemical contexts. In particular, we observed differential ENT1 staining intensities across cortical layers, with notable expression in the cortex granule cell layer. This distinct cortical expression pattern points toward ENT1’s involvement in sensory processing, as layer IV is the main recipient of sensory input from the thalamus, highlighting ENT1’s possible role in sensory information integration and transmission. This layer is densely packed with astrocytes and pyramidal neurons, receives significant thalamic input, and acts as a primary cortical input layer, especially in sensory processing regions such as the somatosensory cortex [26]. Importantly, structural and functional alterations in this area have been observed in psychiatric disorders linked to emotion regulation, including depression, schizophrenia, and anxiety disorders [27]. The high ENT1 expression in the somatosensory cortex may indeed suggest an essential role in emotional processing, particularly through its connections with thalamic circuits. The thalamus serves as a crucial relay station in the brain, transmitting sensory and motor signals to and from the cerebral cortex [28]. The presence of ENT1 in the thalamus suggests its involvement in reward processing, motivation, decision-making, and sleep-wakefulness, as the thalamus plays significant roles in regulating motivated behaviors and thalamocortical neural circuits in sleep and wake cycles [29] .

Moreover, we detected ENT1 expression in the lateral hypothalamic area within the hypothalamus. The lateral hypothalamic area is a neuroanatomical hub that regulates a variety of motivated behaviors, including ingestion and reward-related behaviors, and this population includes functionally diverse neurons that project to different brain regions, thereby performing different functions [30]. Given the critical role of the hypothalamus in regulating appetite, hunger, and satiety, ENT1 may be associated with food intake, energy expenditure, and metabolic states [ 31, 32]. Furthermore, ENT1 is present in the mitochondrial membrane, facilitating the entry of fesylideneuridine (a nucleoside drug) into the mitochondria to induce mitochondrial toxicity [33]. Mitochondrial ENT1 is closely related to energy metabolism, as inhibition of ENT1 enhances the expression of energy-related genes and increases the mitochondrial load and fatty acid metabolism [34]. These findings, together with our findings showing that ENT1’s subcellular localization is mainly in mitochondria, indicate that ENT1 may regulate energy metabolism. Therefore, future studies are warranted to elucidate the roles of ENT1 and adenosine signaling in metabolic functions and the neural circuitry underlying energy metabolism. Notably, our findings revealed ENT1 localization to lysosomes. Previous studies have suggested a functional association between ENT1 and lysosomal membrane protein (CLN3), suggesting that ENT1 contributes to lysosomes [35]. Given the role of lysosomes in protein degradation and autophagy, the observed ENT1-lysosome association raises intriguing questions about its potential involvement in lysosome-mediated processes, such as macromolecule turnover, autophagic flux, or apoptosis [ 36, 37]. While ENT1 may undergo lysosomal degradation, its retention in lysosomes implies a possible functional role that warrants further investigation. Future studies should clarify whether ENT1 directly modulates lysosomal activity or serves as a regulatory component in lysosome-related pathways.

Our findings indicate that ENT1 may be expressed primarily on synapses, which suggests that ENT1 functions to transport intracellular adenosine into the synaptic gap to carry out physiological functions [9]. By modulating adenosine release, ENT1 plays a crucial role in maintaining synaptic transmission and neuronal excitability, contributing to the homeostatic functions of adenosine in the CNS [9]. Nevertheless, some studies have reported that ENT1 may also be localized in astrocytes, where it influences neuronal function indirectly by modulating adenosine signaling [38]. While ENT1 is detectable at low levels in astrocytes [ 39– 41], our cortical data show strikingly limited ENT1 expression in this cell population. This contrasts with its robust neuronal localization, suggesting compartmentalized purinergic signaling mechanisms across cell subtypes and regional differences in the brain. Furthermore, we observed that ENT1 is predominantly expressed in cholinergic, dopaminergic and glutamatergic neurons, supporting previous studies showing that ENT1 regulates acetylcholine, dopamine or glutamatergic release [ 38, 42– 45]. Specifically, inhibition of ENT1’s ability to transport adenosine from the extracellular space prevents the reduction of acetylcholine release [42], and ENT1 regulates dopamine release through the adenosine receptor [38]. Moreover, ENT1 inhibition reduces seizure severity by suppressing glutamatergic neurotransmission [ 43– 45], underscoring its role in cholinergic, dopaminergic, and glutamatergic pathways and its potential as an antiepileptic target. These findings underscore the critical role of ENT1 in neurotransmitter regulation and suggest that ENT1 may serve as a potential target for therapeutic strategies aimed at modulating cholinergic, dopaminergic and glutamatergic signalling in neurological and psychiatric disorders, where these pathways are often dysregulated [46], such as Parkinson’s disease, Alzheimer’s disease, epilepsy, and schizophrenia.

In this study, we first report an increase in ENT1 expression with age in the cerebral cortex, suggesting the potential regulatory roles of ENT1 in aging-related processes. Although our IHC data also revealed a significant increase in ENT1 expression in the hippocampus with age, the western blot analysis and qRT-PCR data did not show such a trend. This apparent discrepancy likely reflects: (1) the dilutional effect of homogenizing anatomically distinct subregions with varying expression patterns and (2) the superior sensitivity of IHC in detecting localized protein distribution compared with that of bulk biochemical methods. Although ENT1 and its role in aging have been widely studied, much of the current research has focused on peripheral diseases. For example, studies have shown that aged mice lacking ENT1 exhibit abnormal bone mineral density [47] and that aging increases ENT1 expression in the carotid body [48]. However, the role of CNS ENT1 in aging-related diseases remains unclear. Further research elucidating the precise mechanisms by which ENT1 contributes to age-related physiological changes will be crucial for developing targeted therapies aimed at restoring adenosine balance in age-related disease states. This study provides a comprehensive analysis of ENT1 expression in the mouse brain, including its regional distribution, cell type specificity, and subcellular localization. All the antibodies were rigorously validated through both isotype controls and secondary antibody controls, with no nonspecific staining observed in these validation experiments. While minimal background staining was noted in the mouse IgG2b isotype control (potentially due to endogenous immunoglobulin interactions in mouse tissue), this staining exhibited distinct morphology and significantly lower fluorescence intensity than did specific MBP and GFAP immunostaining, confirming the high specificity of our primary antibodies.

In conclusion, this study provides an analysis of ENT1 distribution and expression across regions in the mouse brain, emphasizing its neurobiological functions and potential therapeutic relevance. The distinct localization patterns of ENT1, particularly in neuron-dense regions critical for cognitive and sensory functions, underscore its importance in maintaining CNS homeostasis. The link between ENT1 expression and neuronal function further suggests its potential as a therapeutic target for CNS diseases, particularly neurodegenerative and age-related conditions. This research lays essential groundwork for future studies focused on nucleoside transporter systems, aiming to deepen our understanding of the contribution of ENT1 to CNS function and explore its role in the pathology and treatment of neurological disorders.

## Supporting information

25182supplementary_Figures

## Supplementary Data

Supplementary data are available at *Acta Biochimica et Biophysica Sinica* online.
