# Supplementary material for: The expression profile of type 1 equilibrative nucleoside transporter in aged C57BL/6J mouse brain: ENT1 expression in aged mouse brain
Source: Acta Biochim Biophys Sin (Shanghai). 2025 Jul 28;58(4):780–91. doi: 10.3724/abbs.2025127 (PMC13107018; doi:10.3724/abbs.2025127)
Supplement: 25182supplementary_Figures [file 25182supplementary_Figures.docx]

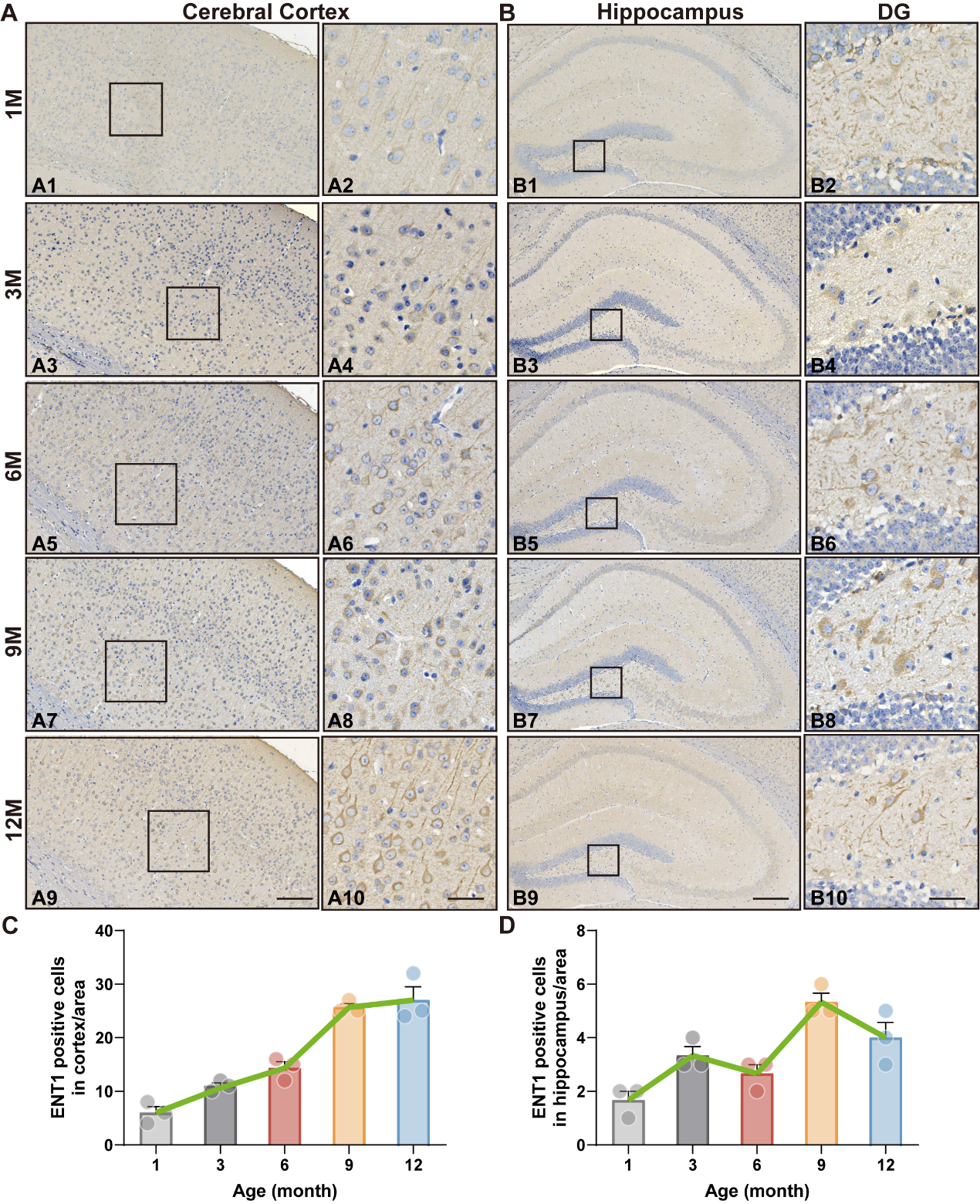


**Supplementary Figure S1. Expressions of ENT1 in the cortex and hippocampus of mice in different age groups** (A) Representative images showing ENT1-positive cells in the cerebral cortex of 1-month-old (1 M; A1−A2), 3-month-old (3 M; A3−A4), 6-month-old (6 M; A5−A6), 9-month-old (9 M; A7−A8), and 12-month-old (12 M; A9−A10) mice via IHC. (B) Representative images showing ENT1-positive cells in the hippocampi of 1 M (B1−B2), 3 M (B3−B4), 6 M (B5−B6), 9 M (B7−B8), and 12 M (B9−B10) mice via IHC. (C) Quantification of ENT1-positive cells per unit area in the cortex of mice in different age groups via IHC. Two-tailed unpaired *t* tests were used: 1 M vs 9 M: *t*_4_ = 14.75, *P* = 0.0001; 3 M vs 9 M: *t*_4_ = 16.63, *P* < 0.0001; 6 M vs 9 M: *t*_4_ = 8.246, *P* = 0.0012; 9 M vs 12 M: *t*_4_ = 0.5121, *P* = 0.6355. (D) Quantification of ENT1-positive cells per unit area in the hippocampus of mice in different age groups via IHC. Two-tailed unpaired *t* tests were used: 1 M vs 9 M: *t*_4_ = 7.778, *P* = 0.0015; 3 M vs 9 M: *t*_4_ = 4.243, *P* = 0.0132; 6 M vs 9 M: *t*_4_ = 5.657, *P* = 0.0048; 9 M vs 12 M: *t*_4_ = 2.000, *P* = 0.1161. The statistical method we used was to count the number of ENT1-positive cells observed under a 40× microscope. Scale bar = 100 μm (A1−A9), 200 μm (B1−B9), and 40 μm (A2−A10, B2−B10). *n* = 3–6 mice per group; *P* < 0.05 indicates significant differences. The data are presented as the mean ± SEM.


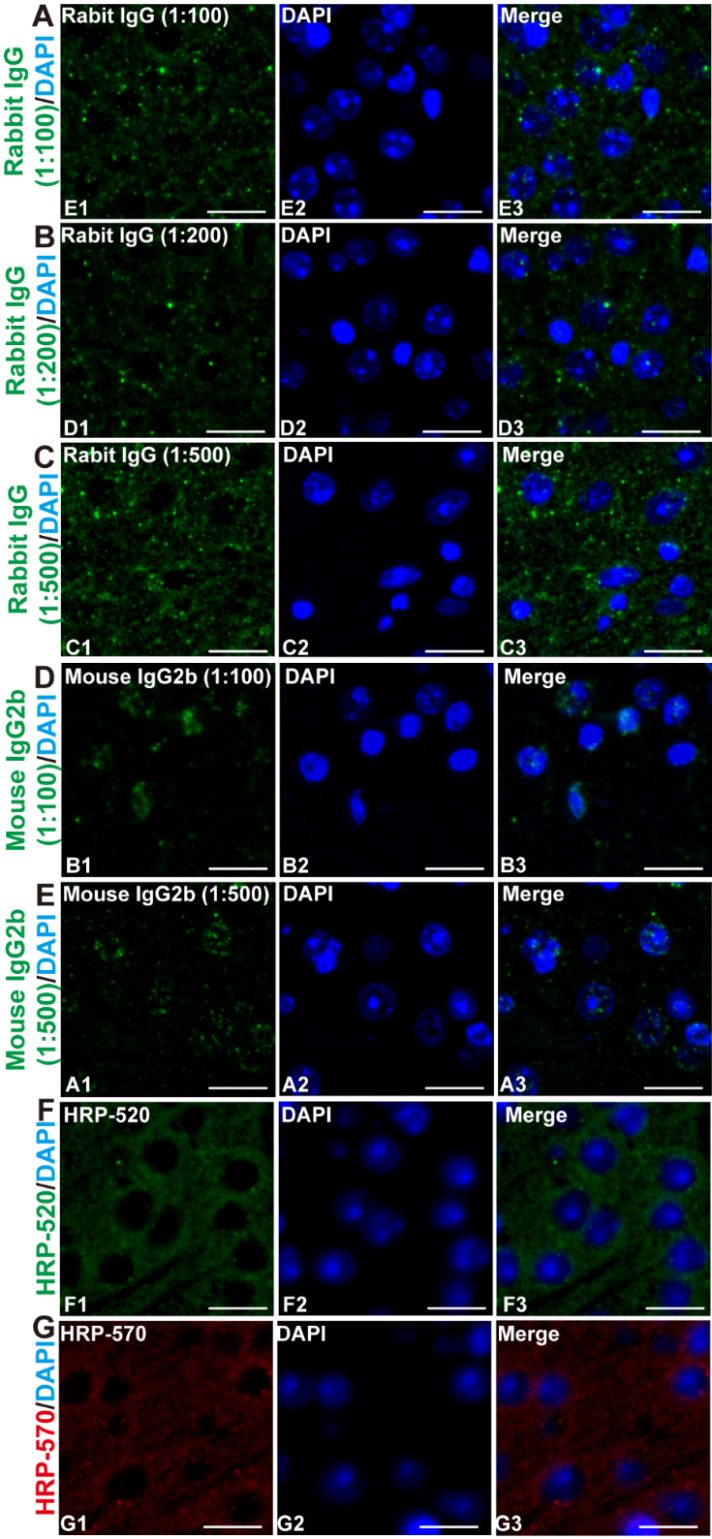


**Supplementary Figure S2. Negative controls for immunofluorescence** **staining** (A−E) Representative images showing the isotype controls for rabbit IgG antibodies at dilutions of (A) 1:100 (for AChE/DAT/VGluT1/LAMP1/TOMM20/COXIV/ENT1), (B) 1:200 (for PSD95), and (C) 1:500 (for Iba-1/MAP2/GAD65/GM130/calnexin). Representative images show the isotype controls for mouse IgG2b antibodies at dilutions of (D) 1:100 (for GFAP) and (E) 1:500 (for MBP). In panels A−E, green fluorescence represents antibodies of isotype controls, and blue fluorescence represents DAPI. (F−G) Representative images showing the negative controls for (F) green fluorescent dyes (TYR-520) and (G) red fluorescent dyes (TYR-570). The blue fluorescence indicates DAPI. Scale bar = 20 μm.
